# Ethanol disrupts hepatocellular lipophagy by altering Rab5-centric LD-lysosome trafficking

**DOI:** 10.1097/HC9.0000000000000446

**Published:** 2024-05-22

**Authors:** Micah B. Schott, Cody N. Rozeveld, Saumya Bhatt, Bridget Crossman, Eugene W. Krueger, Shaun G. Weller, Karuna Rasineni, Carol A. Casey, Mark A. McNiven

**Affiliations:** 1 Department of Biochemistry and Molecular Biology, University of Nebraska Medical Center, Omaha, Nebraska, USA; 2 Department of Biochemistry and Molecular Biology, Division of Gastroenterology and Hepatology, Mayo Clinic, Rochester, Minnesota, USA; 3 Department of Internal Medicine, University of Nebraska Medical Center, Omaha, Nebraska, USA; 4 Department of Veterans’ Affairs, VA-Nebraska-Western Iowa Health Care System, Omaha, Nebraska, USA

## Abstract

**Background:**

Previous reports suggest that lipid droplets (LDs) in the hepatocyte can be catabolized by a direct engulfment from nearby endolysosomes (microlipophagy). Further, it is likely that this process is compromised by chronic ethanol (EtOH) exposure leading to hepatic steatosis. This study investigates the hepatocellular machinery supporting microlipophagy and EtOH-induced alterations in this process with a focus on the small, endosome-associated, GTPase Rab5.

**Methods and Results:**

Here we report that this small Ras-related GTPase is a resident component of LDs, and its activity is important for hepatocellular LD-lysosome proximity and physical interactions. We find that Rab5 siRNA knockdown causes an accumulation of LDs in hepatocytes by inhibiting lysosome dependent LD catabolism. Importantly, Rab5 appears to support this process by mediating the recruitment of early endosomal and or multivesicular body compartments to the LD surface before lysosome fusion. Interestingly, while wild-type or a constituently active GTPase form (Q79L) of Rab5 supports LD-lysosome transport, this process is markedly reduced in cells expressing a GTPase dead (S34N) Rab5 protein or in hepatocytes exposed to chronic EtOH.

**Conclusions:**

These findings support the novel premise of an early endosomal/multivesicular body intermediate compartment on the LD surface that provides a “docking” site for lysosomal trafficking, not unlike the process that occurs during the hepatocellular degradation of endocytosed ligands that is also known to be compromised by EtOH exposure.

## INTRODUCTION

Alcohol-associated liver disease is the leading cause of alcohol-associated mortality and present within 90% of heavy drinkers.^1^ Early stages of alcohol-associated liver disease are marked by hepatic steatosis, which is the aberrant accumulation of lipid droplets (LDs) within hepatocytes, the parenchymal cells of the liver. EtOH-induced steatosis is caused in part by a reduction in LD catabolism by lipophagy, which is the selective autophagy of LDs.^2^ During this process, LDs are selected for degradation within acidic lysosomes harboring lysosomal acid lipase (LAL), which functions as the principal lipase in lipophagy.^3^

Although lipophagy was first described as an autophagosome-dependent catabolism of LDs (termed macrolipophagy), it is now recognized that LD-lysosome trafficking can occur independent of canonical autophagosome targeting.^4,5^ The latter process, termed microlipophagy, was first described in yeast but has since been shown to occur in mammalian cells including macrophages and hepatocytes.^4,6–10^ Although ethanol exposure has been shown to diminish lipophagy,^11^ the impact of EtOH on macro versus microlipophagy in hepatocytes remains poorly defined but may rely on the dysregulation of various Rab GTPases that function in membrane trafficking. For example, EtOH can directly inhibit the GTPase activity of Rab7, a late endosomal Rab GTPase, which impacts the downstream trafficking of LDs into the lysosomal lumen.^11,12^ However, early upstream events that select LDs for lipophagy, particularly autophagy-independent microlipophagy, are poorly understood.

LDs are host to several different Rab GTPases that are otherwise recognized as key regulators of vesicular trafficking. Not surprisingly, some of these LD-resident Rab GTPases play parallel roles in lipophagy. This includes Rab10, which recruits autophagosomes to LDs and is thought to facilitate autophagic membrane extension around the LD surface.^13,14^ Rab7 is also present on LDs and has been shown previously to facilitate LD interactions with late endosomes and multivesicular bodies.^15^ In activated hepatic stellate cells, Rab25 is induced by reactive oxygen species to recruit autophagosomes to LDs for lipophagy.^16^ Further, Golgi-localized Rab40 in *Drosophila* interacts with its GTPase activating protein TBC1D22 to regulate Lamp1-dependent lipophagy.^17^

Rab5 is an early endosome GTPase that has long been reported as a LD-resident protein based on proteomic studies.^18–23^ Rab5 localization to LDs appears to require its C-terminal CAAX domain, as prenylation-deficient mutants are largely absent from LDs.^24^ Earlier studies in HeLa cells suggest that Rab5 GTPase activity regulates LD recruitment of early endosomes positive for transferrin receptor and early endosome antigen 1 (EEA1), an Rab5 effector.^25^ Given the importance of endosomal vesicles on microautophagy of organelles,^26^ Rab5 may represent an attractive early target for directing LDs to lysosomes through microlipophagy. Interestingly, a recent screen identified Rab5 as a candidate gene involved in macrophage cholesterol efflux, a process that relies heavily on lipophagy.^10^ However, the role of Rab5 in hepatocellular lipophagy is unclear, nor has the impact of EtOH on Rab5 been explored.

In the present study, we sought to define the impact of EtOH on macro versus microlipophagy in primary hepatocytes from rats fed a chronic EtOH diet. This study is based on our preliminary findings that EtOH-damaged hepatocytes show little to no interactions or proximity between LDs and endolysosomes: a phenotype that is mimicked in cells with reduced Rab5 levels. Subsequently, we find a key role for the Rab5 GTPase in directing LDs toward a transferrin-positive early endosome compartment. Ethanol promotes the accumulation of Rab5-positive vesicles around LDs, likely by inhibiting downstream lysosomal fusion through the targeting of Rab7. Overall, these studies provide mechanistic insights into ethanol insult and hepatocellular microlipophagy as a trafficking pathway similar to that of endosomal vesicles.

## METHODS

### Cell culture and reagents

Primary rat hepatocytes were isolated from male Wistar rats pair-fed with Lieber-DeCarli control or ethanol liquid diet for 6 weeks and cultured in William’s E Medium (5% Fetal Bovine Serum [FBS]) as described.^27^ All animals received humane care in accordance with the guidelines established by the American Association for the Accreditation of Laboratory Animal Care, and animal protocols were approved by the Institutional Animal Care and Use Committee at the University of Nebraska Medical Center. The VA13 cells are HepG2 human hepatoma cells stably expressing alcohol dehydrogenase. VA13 cells were maintained in DMEM supplemented with 10% FBS, Pen/Strep, and Zeocin antibiotic. The β-actin antibody (A2066), DGAT1 inhibitor PF-04620110 (PZ0207), DGAT2 inhibitor PF-06424439 (PZ0233), Oleic Acid (OA) (O1008), and Oil Red O (O0625) were from Sigma-Aldrich. The monodansylpentane was from Abgent (SM1000a). The PLIN2 antibody (B3121) was from LS Biosciences. The LC3 antibody (NB600-1384) was from Novus. The Lamp1 antibody (1D4B) was from the Developmental Studies Hybridoma Bank at the University of Iowa (Iowa City, IA). The LAL inhibitor LALi (6098) was from Tocris. The C12-BODIPY (FL: D3822; 558/568: D3835) and LysoTracker Deep Red (L12492) were from Thermo Fisher Scientific. The EGFP-LC3 was a gift from K. Kirkegaard (Stanford, CA; Addgene plasmid 11546). The GFP-Rab5 was a gift from Marci Scidmore (Addgene plasmid # 49888). The GFP-Rab5(Q79L) and GFP-Rab5(S24N) were gifts from Sergio Grinstein (Addgene plasmids #35140 and 35141, respectively). Cells were transfected at 60%–80% confluency using Lipofectamine 2000 transfection reagent (Thermo Fisher Scientific) according to the manufacturer’s instructions.

### Fluorescence microscopy

The cells were washed in PBS and fixed in 3% formaldehyde as described previously. To label LDs, fixed samples were washed in 60% isopropanol for 30 seconds, 60% Oil Red O (ORO) solution (5 mg/mL in isopropanol) for 2.5 minutes, and then washed in 60% isopropanol for an additional 30 seconds. The images were acquired using a Zeiss LSM 800 confocal microscope with a 40× oil objective lens (NA=1.4). LD measurements were done using ImageJ software. Quantification of LD size, number, and total area per cell was done using ImageJ software. To quantify LD-lysosome trafficking, cells were first labeled with 7.5 µM BODIPY-(558/568)-C12 lipid for 2 hours, then washed 2× in HBSS and placed into regular medium for 24 hours in DMSO or LAListat (50 µM). Cells were fixed and immunolabeled with a LAMP1 antibody. Quantification of lysosomal lipid accumulation was done by manual counting for Lamp1 immunostaining, or using ImageJ for lysotracker staining where lysosomes were segmented and the fluorescence intensity of the lipid was quantified within the segmented lysosome area (Schulze, 2020 #554).^4^

### Electron microscopy

AML12 cells were plated on glow-discharged, carbon-coated coverslips in full media with pen/strep and 10% FBS. The next day, the media was exchanged to full media with pen/strep and 10% FBS and supplemented with 200 μM oleate and cultured for another 24 hours. The cells were washed 3× with HBSS (+Ca+Mg) and then incubated for 60 minutes in HBSS (+Ca+Mg) in the presence of the 15 nm gold-conjugated transferrin (Cytodiagnostics). After 60 minutes, the cells were washed extensively in HBSS (+Ca+Mg) and processed for transmission electron microscopy. Briefly, for standard transmission electron microscopy, cells on carbon-coated coverslips were rinsed in 37°C HBSS and fixed with 37°C primary fixative (100 mM cacodylate, pH 7.4, 60 mM sucrose, 2.5% glutaraldehyde) for 1 hour at room temperature, rinsed 3 times with washing buffer (100 mM cacodylate, pH 7.4, 200 mM sucrose), then fixed in the secondary fixative (50 mM cacodylate, pH 7.4, 100 mM sucrose, 1% OsO_4_) for 1 hour at room temperature, rinsed 3 times in water, and fixed in 1% uranyl acetate in water for 1 hour at room temperature. Samples were then dehydrated in a graded ethanol series, embedded in Quetol 651 (Ted Pella), and polymerized in a 65°C oven overnight. After removal from the oven, the coverslip was removed from the bottom of the sample, the block trimmed down to a trapezoid 1 mm wide at the base, and 100-nm-thin sections were cut and subsequently viewed on a Jeol 1400 transmission electron microscope (Jeol Ltd).

### LD isolation

LDs were isolated from VA13 cells grown to 90% confluency in 5 × 15 cm dishes and loaded with OA (150 µM, 16 h), or from 50 million freshly isolated primary hepatocytes from rat liver. Using a protocol adapted from 2 previous studies,^28,29^ cells were incubated in a hypotonic lysis medium, then lysed using a Dounce homogenizer. The postnuclear supernatant was placed at the bottom of a 30%–0% OptiPrep density gradient (Sigma D1556). Following a 30-minute 17,200 rpm spin, the floating fat layer was collected and washed for subsequent western blot analysis. Protein levels were normalized to PLIN2 as a loading control.

### siRNA knockdown

Cells were grown to 50%–80% confluency before transfection using Lipofectamine RNAiMAX (13778-150, Thermo Fisher). siOnTARGET pools (Horizon Discovery) targeting human Atg5 (L-004374-00), Fip200 (L-021117-00), Rab5 (:L-004009-00), and Rab7 (L-010388-00), or a nontargeting control siRNA (D-001210-01), were used for knockdowns of 72 hours.

### GFP immunoprecipitation

For immunoprecipitation of GFP-tagged Rab5, GFP-Trap agarose beads (Chromotek) were used according to the manufacturer’s instructions. In brief, cells were plated to 3 wells of a 6-well plate and transfected with GFP-Rab5 at 60%–80% confluency. Cells were lysed in 200 μL lysis buffer (10 mM Tris/Cl pH 7.5, 150 mM NaCl, 0.5 mM EDTA, 0.5% NP40), then diluted in 300 μL dilution buffer (10 mM Tris/Cl pH 7.5, 150 mM NaCl, 0.5 mM EDTA). A 25 μL bead slurry was equilibrated 3× in 500 μL dilution buffer, then mixed with diluted lysate for 1 hour, rotating at 4°C. Beads were washed 3× in wash buffer (10 mM Tris/Cl pH 7.5, 150 mM NaCl, 0.5 mM EDTA, 0.05% NP40), then resuspended in 80 μL of 2× sample buffer for western blot analysis.

### GST pulldown

Pulldown of active Rab5 was achieved using a GST fusion protein with the Rab5-binding domain (R5BD) of Rabaptin5 (residues 739–862). The pGEX-4 T-2/Rabaptin-5:R5BD plasmid was a gift from Dr Guangpu Li (University of Oklahoma). Fusion proteins were expressed in *Escherichia coli* BL21(DE3) pLysS-competent cells (Invitrogen) and purified using glutathione Sepharose 4B beads (GE Healthcare) according to the manufacturer’s instructions. GST pulldown was performed as previously described (PMID: 25800849). Cells were lysed in lysis buffer (25 mM HEPES-KOH pH 7.4, 100 mM NaCl, 5 mM MgCl_2_, 0.1% NP40, 10% glycerol, 1 mM dithiothreitol, and protease inhibitor cocktail) and incubated with GST-Rabaptin(R5BD) for 30 minutes at 4°C on a rotation mixer and washed in lysis buffer lacking detergent (25 mM HEPES-KOH pH 7.4, 100 mM NaCl, 5 mM MgCl_2_, 10% glycerol, 1 mM dithiothreitol, and protease inhibitor cocktail).

## RESULTS

### Chronic EtOH attenuates LD-lysosome transport in hepatocytes

**FIGURE 1:**
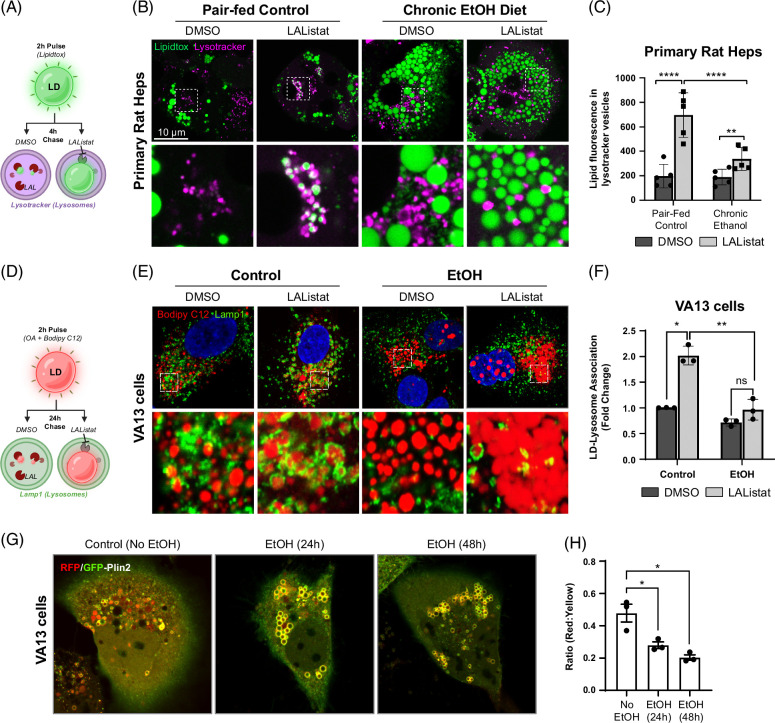
Chronic EtOH consumption blocks LD-lysosome trafficking and increases Rab5-LD association. (A) Cartoon depiction of the lipid pulse-chase experiments used to assess LD trafficking in primary hepatocytes. (B) Confocal micrographs showing lipidtox-stained LDs (green) and lysotracker-stained lysosomes (magenta) in primary hepatocytes from pair-fed control and chronic EtOH-fed rats. Note the accumulation of LDs within lysosomes after 24-hour treatment with 50 μM LAListat in pair-fed control cells, a process that is diminished significantly in hepatocytes from EtOH-fed rats despite an overall abundance of LDs. (C) Quantification of lysosomal lipid accumulation from n=5 independent experiments, ~200 cells per experiment. (D) Cartoon depiction of pulse-chase assay using fluorescent bodipy C12 lipid performed in VA13 cells. (E) Confocal micrographs showing bodipy-stained LDs (red) and Lamp1-immunolabeled lysosomes (green) in VA13 hepatocytes treated with 0 mM (control) versus 100 mM EtOH for 48 hours. Note that LDs accumulate within lysosomes after 24-hour treatment with 50 μM LAListat in control cells, but this interaction is reduced markedly in EtOH-fed cells. (F) Quantification of lysosomal lipid accumulation from VA13 cells. (G) Confocal live-cell imaging of VA13 cells expressing a GFP-RFP-Plin2 lipophagy reporter that shifts from yellow to red when entering the acidic lysosome. Note the abundant red-only puncta indicating normal lipophagy in control cells (no EtOH). The predominant number of red puncta dissipate over 24- and 48-hour treatment with 100 mM EtOH compared to control cells. (H) Graph depicting the ratio of “red-only” puncta as a function of yellow (green+red) fluorescence following 0, 24, and 48-hour treatment with 100 mM EtOH in VA13 cells. Asterisks denote statistical significance by one-way ANOVA and Tukey post-hoc test (*p < 0.05; **p < 0.01). Graphs depict mean and SD. Abbreviations: LD, lipid droplet; OA, Oleic Acid.

In our initial findings from primary hepatocytes isolated from EtOH-fed rats, we observed a marked attenuation in the number of interactions between LDs and lysosomes. To pursue this finding further, we made generous use of an inhibitor of the lysosome acid lipase (LAListat) which prevents lipolysis by this organelle resulting in an accumulation of newly engulfed LDs in the lysosome lumen (Figure 1A). This simple approach allows one to measure changes in the lipophagic process over time, and during experimental manipulations such as EtOH exposure, as engulfed LDs are not degraded. As displayed in Figure 1B, hepatocytes isolated from control fed rat livers, and treated with LAL for 4 hours before confocal imaging reveal >2 fold more lysosome-internalized LDs than hepatocytes isolated from EtOH-fed rats despite a substantial elevation in total LD content caused by EtOH damage (Figure 1B, C). Parallel studies of LD-engulfment by lysosomes were also performed in a widely used HepG2 hepatoma cell line stably expressing alcohol dehydrogenase (VA13 cells) exposed to 100 mM EtOH for 48 hours. In this assay, cells were first pulse-labeled with fluorescent Bodipy C12 to label LDs (7.5 μM, 2 h), then washed and chased in regular medium containing DMSO or LAListat (50 μM, 24 h) as illustrated in Figure 1D. Consistent with the findings in primary hepatocytes, there was a striking decrease in the number of LDs observed within the lumen of lysosomes in the EtOH-treated VA13 cells (Figure 1E, F). Further, the proximity between LDs and lysosomes in the EtOH-treated cells appeared markedly increased as if any attraction between the 2 organelles had been lost, suggestive of a defect in organelle trafficking. The interaction between these organelles was also measured using a previously described RFP-GFP-Plin2 lipophagy reporter^4,15^ in which the Plin2 protein targets the RFP-GFP fluorophores to the LD surface as overlapping yellow fluorescence. Entry of the LDs into the acidic environment of the lysosomal lumen quenches the GFP-fluorescence resulting in red puncta depicting LDs within the lysosome. Thus, changes in lysosome-LD interactions by EtOH exposure can be compared and quantitated. As depicted in Figure 1G, control VA13 cells display numerous red-emitting LDs within lysosomes. A stepwise decrease in “red-only” puncta is observed following 24 and 48 hours exposure to 100 mM EtOH, indicating a time-dependent, marked decrease, in lipophagy (Figure 1H).

**FIGURE 2:**
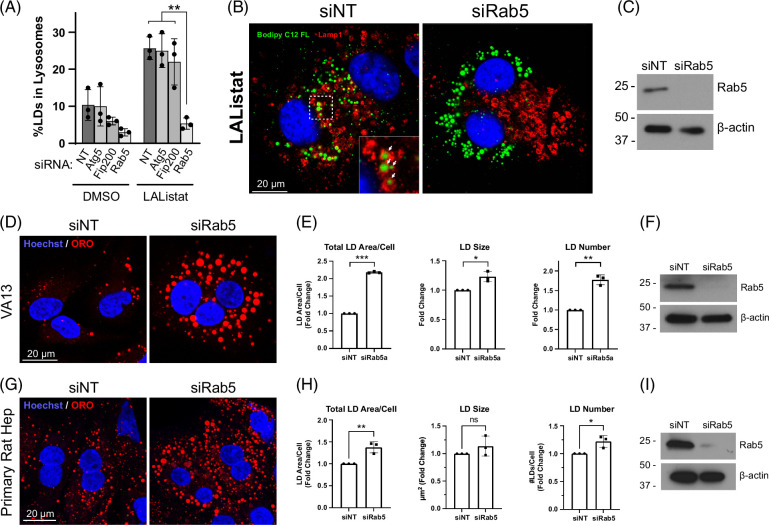
Lipid droplet trafficking to lysosomes is dependent on Rab5 but not the macroautophagy machinery. (A) Effects of siRNA knock down of Rab5 and 2 essential canonical autophagy proteins (ATG5 and FIP200) in VA13 cells on the percentage of total LDs internalized by lysosomes using the bodipy pulse-chase assay as described in Figure 1D. The bar graph depicts a marked reduction of LD accumulation in lysosomes following LAListat treatment in siRab5-treated cells that, in comparison, is unaltered in cells with reduced ATG5 or FIP200. (B) Confocal micrographs of LAListat-treated cells show robust LD engulfment by LAMP1-labeled lysosomes in control siNT cells as indicated by white arrows. Conversely, LD-Lamp1 interactions are markedly reduced in Rab5-depleted cells. (C) Representative western blot confirms knockdown of Rab5. Note that representative micrographs and western blot of Atg5 and Fip200 knockdown are depicted in Supplemental Figure S1, http://links.lww.com/HC9/A892. (D) Oil Red O-stained VA13 cells showing the marked increased LD area following Rab5 siRNA knockdown. (E) Bar graphs depict quantification of the increase in total LD area/cell, LD size (μm^2^), and LD number/cell following a Rab5 knockdown. (F) Representative western blot showing Rab5 knockdown efficiency. (G) Rab5 knockdown in primary rat hepatocytes increases ORO-stained LD content. (H) Quantification revealed significant increases in total LD area/cell and LD number/cell. (I) Representative western blot showing Rab5 knockdown efficiency. Asterisks denote statistical significance by *t* test (*p < 0.05; **p < 0.01). Graphs depict mean and SD. Abbreviation: LD, lipid droplet.

Our previous study demonstrated that an active lipid transfer can occur at contact sites between LDs and lysosomes, facilitating lipid catabolism in hepatocytes.^4^ Furthermore, this process represented microlipophagy as it occurred independent of macroautophagy and chaperone-mediated autophagy machineries.^30^ Based on the findings described above in Figure 1, we sought to test whether microlipophagic versus macrolipophagic processes were disrupted by EtOH exposure. To this end, the contribution of macroautophagic machinery to LD-lysosome lipid interactions was assessed in VA13 cells using siRNA knockdown of Atg5 and Fip200, both proteins are known to be essential in the formation of a nascent canonical phagophore. In addition, we tested the contribution of endocytic vesicles to this process using siRNA knockdown against the early endosome protein Rab5, which has previously been shown to reside on LDs.^25^ Cells were loaded with fluorescent lipid and chased in DMSO versus LAListat as depicted in Figure 1D. While cells chased in DMSO media showed a modest accumulation of Bodipy C12-labeled LDs in LAMP1 immunolabeled lysosomes, LAListat-treatment caused a robust accumulation of LDs within lysosomes in both nontargeting control siRNA-treated cells as well as in siAtg5 and siFip200 cells (Figure 2A, Supplemental Figure S1A, B, http://links.lww.com/HC9/A892). Importantly, this increase was almost completely abolished in cells depleted of Rab5 (Figure 2A, B). Further, these organelles appeared partitioned to distinct regions of the cell showing little to no affinity toward each other. Indeed, while lysosomes appeared to gather at the cell center near the centrosome in these altered cells, LDs were often located in the opposing periphery of the cytoplasm. These results suggest that the macroautophagy machinery, particularly Atg5 and Fip200, are not required for LD-lysosome transport while this process utilizes Rab5-dependent endosome trafficking.

The results above suggest that endosome-based microlipophagy plays a prominent role over autophagosome-based macrolipophagy in hepatocytes, in agreement with our previous work.^4^ To explore this further, we analyzed the relative frequency of LD association with lysosomes, autophagosomes, and autolysosomes in VA13 cells transfected with the autophagosome marker GFP-LC3 that were then stained with the LD marker monodansylpentane as well as lysotracker deep red. As shown in Supplemental Figure S1C, http://links.lww.com/HC9/A892, several instances of LD-lysosome association were found lacking the presence of LC3, suggesting these vesicles are not autophagosomes or autolysosomes. LD association with lysosomes (lysotracker only), autophagosomes (GFP-LC3 only), and autolysosomes (lysotracker+LC3) was quantified across ~30 cells treated 24 hours with DMSO versus LAListat. As shown in Supplemental Figure S1, http://links.lww.com/HC9/A892, LD-lysosome association was significantly higher than that of autophagosomes and autolysosomes under both conditions. Similar results were found in Hep3B hepatocytes (Supplemental Figure S1D, http://links.lww.com/HC9/A892), as well as AML12 and Huh7 hepatocytes (not shown). Taken together, these findings further support the notion that LDs are directly transported through the cytoplasm toward an endosomal compartment, even in the absence of an autophagosome intermediate.

### Rab5 influences LD trafficking to lysosomes

Microautophagy is the trafficking of cytosolic cargo directly into endolysosomes independent of an autophagosome intermediate. As Rab5 is well known to direct endosome transport^31,32^ and is a prominent resident component of the LD proteome in various cell types including hepatocytes and adipocytes,^18,19,21–23,33,34^ we tested if this regulatory GTPase is present on hepatocyte LDs and plays a role in LD catabolism. First, we performed siRNA knockdown of Rab5 and quantified cellular LD content in both VA13 cells (Figure 2D) and primary rat hepatocytes (Figure 2G). Quantification revealed a marked ~2-fold increase in total LD area per cell and LD number in the knock down VA13 cells (Figure 2E) with similar findings from the primary cells (Figure 2H). Representative western blots confirmed efficient knockdown in both VA13 cells (Figure 2F) and primary rat hepatocytes (Figure 2I).

Given the findings that siRNA knockdown of Rab5 has on LD levels, it became important to define if this represented a substantial defect in overall LD catabolism or LD synthesis. To this end, inhibitors of the LD synthesis enzymes diacylglycerol acyltransferase 1 and 2 (DGAT1, DGAT2) were utilized in cells treated with nontargeting siRNA (siNT) versus siRab5. This allowed us to test if cells with altered Rab5 activity continued to accumulate LDs even when nascent synthesis is reduced. Cells were loaded with oleic acid (150 μM, 5 h) to induce LD formation, then washed and chased an additional 24 hours in regular medium containing DGAT1 and 2 distinct inhibitors (PF-04620110 and PF-06424439). As shown in confocal images and quantification in Supplemental Figure S2, http://links.lww.com/HC9/A892, ~80% of LD content was lost/catabolized during the 24-hour chase period in control siNT cells, whereas only <30% of LDs were utilized in the Rab5-depleted cells. These findings are consistent with the premise that Rab5 activity mediates hepatocellular LD catabolism rather than synthesis.

**FIGURE 3:**
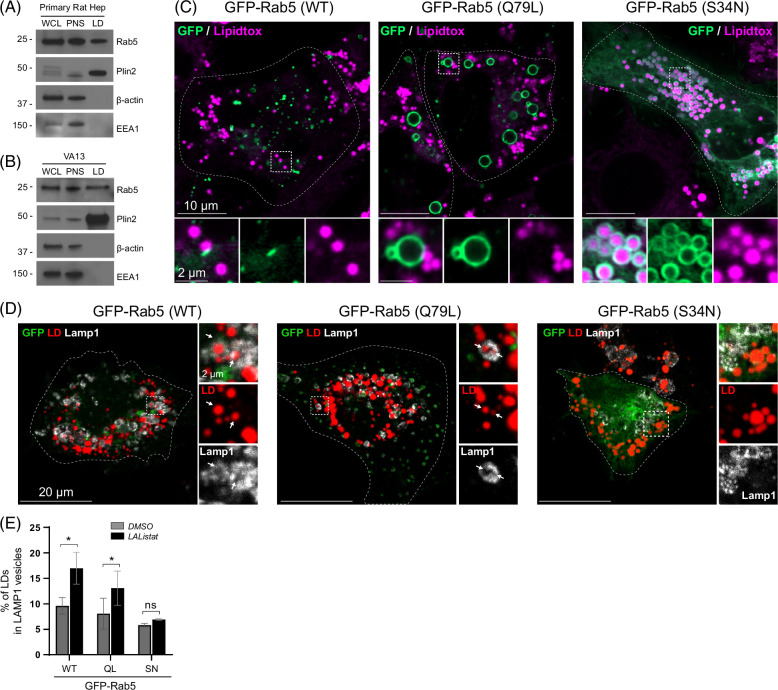
Rab5 localizes to hepatocellular LDs and regulates LD abundance. (A) Western blot of LDs isolated from primary rat hepatocytes or (B) VA13 hepatoma cells showing the Rab5 abundance on LDs. Note the lack of the early endosome marker and Rab5 effector EEA1. WCL is whole-cell lysate. PNS is postnuclear supernatant. (C) Confocal micrographs depicting the localization of wild-type (WT) GFP-Rab5 that appears as punctate associated with lipidtox-stained LDs. The constitutively active GFP-Rab5 form (Q79L) induces the formation of enlarged endosomes as described previously,^35^ while GTPase defective GFP-Rab5 form (S34N) showed a substantial affinity to LDs. (D) Representative confocal micrographs showing Bodipy-stained LDs (red), and Lamp1-immunolabeled lysosomes in VA13 cells expressing GFP-Rab5 wild-type (WT), constitutively active GFP-Rab5 (Q79L), or dominant-negative GFP-Rab5 (S23N) mutants and treated with either DMSO versus LAListat. Note the significant increase in LD-lysosome association following LAListat in cells expressing WT and QL Rab5, but this is absent in cells expressing the dominant negative SN mutant. (E) Graph representing quantitation of LD trafficking to lysosomes under the conditions described in (D). Abbreviation: LD, lipid droplet; WT, wild-type.

Next, we sought to further characterize the association between Rab5 and LDs. In agreement with previous proteomic studies, Rab5 was detected in purified LDs isolated by density gradient centrifugation in both primary rat hepatocytes (Figure 3A) and VA13 cells (Figure 3B).Surprisingly, the presence of the EEA1 was not detected. Subsequently, using Airyscan confocal microscopy, the localization of 3 different Rab5 GFP plasmids in VA13 cells was assessed and included wild-type (WT) GFP-Rab5, as well as constitutively active GFP-Rab5 (Q79L) and dominant negative GFP-Rab5 (S34N). As shown in Figure 3C, both WT Rab5 and the constitutively active (Q79L) GFP-Rab5 mutant proteins were found in contact with lipidtox-stained LDs. The GFP-Rab5(Q79L) vesicles represent enlarged, hyper-fused endosomes as reported.^36,37^ In contrast, the dominant-negative GFP-Rab5 mutant (S34N) readily localized to LDs, indicating that the Rab5 GTPase activity alters its affinity for the LD surface.

To test the role of Rab5 GTPase activity in LD-lysosome trafficking, VA13 cells were transfected to express either WT, constitutively active (Q79L), or dominant-negative (S34N) variants GFP-Rab5. Cells were first lipid loaded with 150 μM OA for 16 hours, washed, and treated for an additional 24 hours with DMSO versus LAListat before fixation and immunolabeling with a Lamp1 antibody. As shown in Figure 3D, E, LD-lysosome association was increased in LAListat-treated cells expressing either WT or Q79L GFP-Rab5. However, LD-lysosome interactions were blocked in cells expressing the dominant-negative S34N Rab5 mutant. These results suggest that active Rab5 plays a key role in the intimate copositioning of these organelles to facilitate the subsequent microlipophagic process.

**FIGURE 4:**
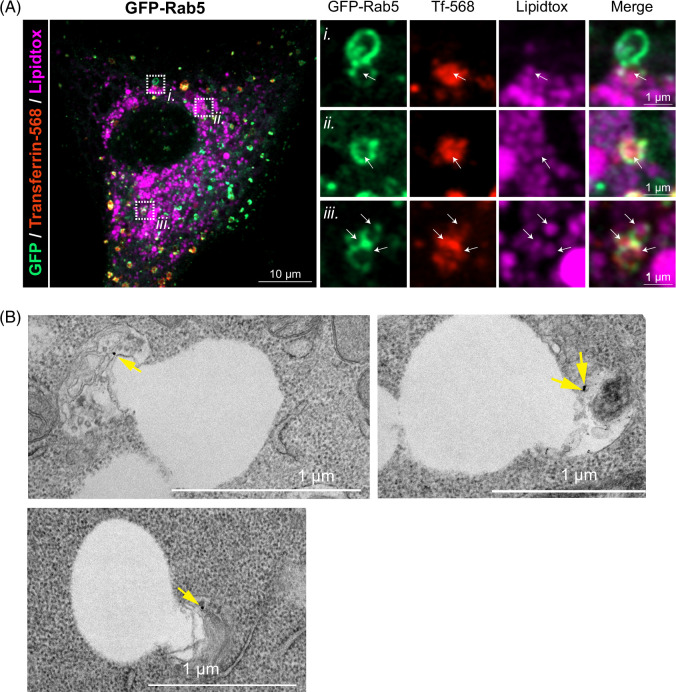
Lipid droplet engulfment by Rab5-associated early endosomes. (A) VA13 cells expressing GFP-Rab5 were incubated 30 minutes with a fluorescent early endosomal cargo transferrin ligand (Tf-568, red) and lipidtox LD stain (magenta). Cells expressing wild-type GFP-Rab5 show engulfment of lipidtox-stained LDs within Rab5 vesicles also positive for transferrin ligand. White arrows in enlarged inlays depict LDs within these vesicles. LD-endosome contacts in cells expressing Q79L and S34N Rab5 are shown in Supplemental Figure S3, http://links.lww.com/HC9/A892. (B) Electron micrographs showing partial LD engulfment into transferrin-positive organelles in AML12 hepatocytes starved in HBSS 1 hour in the presence of 15 nm gold-transferrin. Yellow arrows depict gold spots indicating transferrin-positive spots. Abbreviation: LD, lipid droplet.

To test further whether Rab5 localizes to LDs independently or through an early endosomal compartment, VA13 cells expressing GFP-Rab5 were incubated with 25 μg/ml of a fluorescent transferrin ligand (Tf-568) for 30 minutes before fixation to label the early endosomal compartments. LDs engulfed by GFP-Rab5 (Figure 4A, see also Supplemental Figure 3, http://links.lww.com/HC9/A892) were positive for transferrin ligand, suggesting LD contact with an early endosome compartment. The interaction between LDs and transferrin-positive endosomes was further confirmed by transmission electron microscopy of AML12 hepatocytes. AML12 cells were first loaded with 200 μM OA overnight, then washed in HBSS and loaded with 15 nm gold-labeled transferrin for 1 hour. Shown in Figure 4B are multiple examples of LDs interacting with transferrin-positive endosomes (see also Supplemental Figure 4, http://links.lww.com/HC9/A892). Taken together, these observations support the premise that Rab5 functions to recruit early endosomes to LDs to facilitate lipophagic catabolism in hepatocytes.

### Chronic EtOH exposure increases Rab5 accumulation on LDs

**FIGURE 5:**
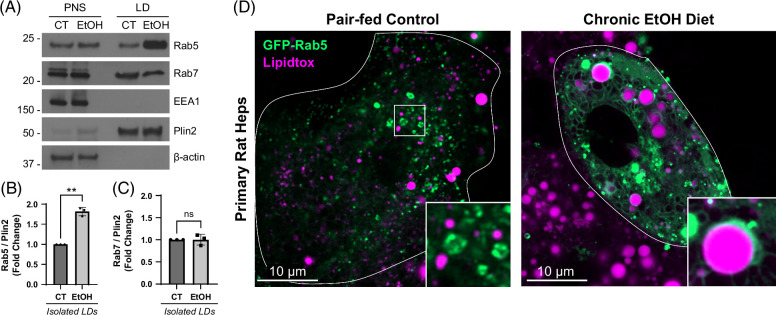
Chronic EtOH exposure increases the association of Rab5 with LDs. (A) Western blot of biochemically isolated LDs showing increased Rab5, but not Rab7, on LDs from chronic EtOH-fed rat hepatocytes versus control. (B, C) Graphs depicting quantification of Rab5, Rab7, and Plin2 levels from western blots of biochemically isolated LDs from n=3 experiments. (D) Confocal live-cell imaging showing the distribution of GFP-Rab5 in hepatocytes from control versus EtOH-fed rats. Note the accumulation of Rab5 on LDs from EtOH-fed rat hepatocytes. Asterisks denote statistical significance by *t* test (*p<0.05; **p<0.01). Graphs depict mean and SD. Abbreviation: LD, lipid droplet.

As the reduction of LD-lysosome interactions in EtOH-damaged hepatocytes mimicked that observed in cells with altered Rab5 expression or activity (Figures 1C, 2A), it was important to test if EtOH might alter the affinity of Rab5 itself for the LD surface. First, western blot analysis was performed on purified LDs from hepatocytes isolated from CT versus EtOH fed rats. Importantly, a >50% enrichment of Rab5 was observed on LDs in response to chronic EtOH diet (Figure 5A, B). As a comparison, there was no observable difference in the levels of the late endosome/MVB (multivesicular body) GTPase Rab7 on LDs between CT versus EtOH rats (Figure 5C). These findings are consistent with a recent unbiased LD proteomic study in hepatocytes showing elevated Rab5, but not Rab7, on LDs from EtOH-fed rats.^23^ To support these biochemical observations, we performed confocal microscopy of primary rat hepatocytes from CT versus EtOH-fed rats transfected with GFP-Rab5. As shown in Figure 5D, the subcellular distribution of Rab5 was markedly altered in hepatocytes from EtOH-fed rats as these cells displayed an increased association of Rab5 with LDs, and in addition, a marked distribution to a membranous network resembling the endoplasmic reticulum. These findings are consistent with the western blotting analysis of purified LDs and support the notion that chronic EtOH causes an accumulation of Rab5 on hepatocellular LDs.

**FIGURE 6:**
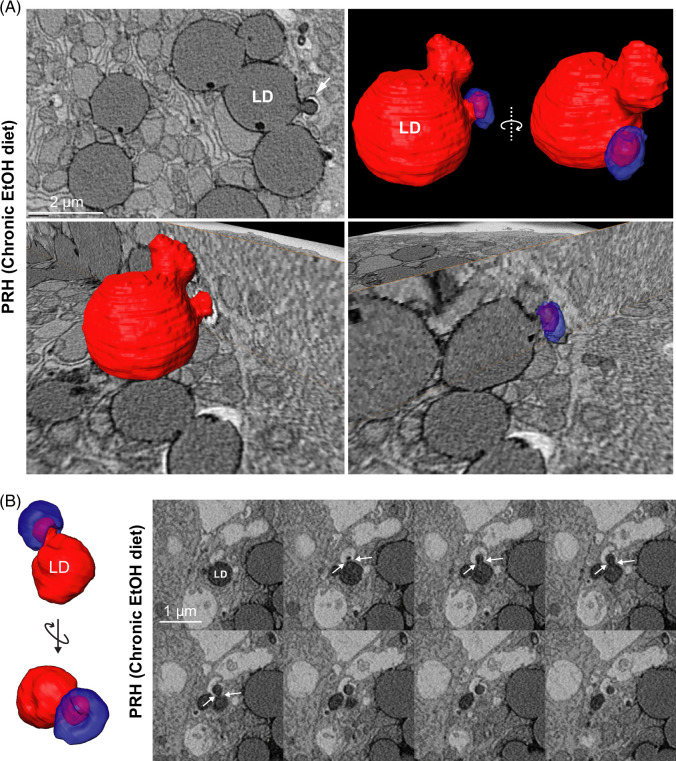
Examples of LD-vesicle engulfment in chronic EtOH-fed hepatocytes. SBF-SEM of primary hepatocytes from EtOH-fed rats showing instances of LD engulfment into intracellular vesicles. (A) 3D reconstruction showing a larger-sized LD (red) ~2 μm in diameter seemingly undergoing piecemeal lipid engulfment into a vesicle (blue) ~500 nm in size. (B) 3D reconstruction showing a smaller, ~500 nm LD undergoing engulfment into similarly sized vesicle. Abbreviations: LD, lipid droplet; SBF-SEM, serial blockface scanning electron microscopy.

The finding that chronic EtOH increased Rab5 abundance on LDs was surprising given that EtOH blocks LD-lysosome engulfment (see Figure 1). To further explore the interaction of LDs with endosomes, we employed serial blockface scanning electron microscopy in primary hepatocytes from chronic EtOH-fed rats. This technique allows for high-resolution electron microscopy in 3D, allowing us to dissect LD-endosome interacting regions with unprecedented detail. Using this approach, we found prominent examples of LDs (pseudocolored red) interacting with intracellular vesicles ~500 nm in diameter (pseudocolored blue). These LD-vesicle interactions seemed to resemble a form of piece-meal lipophagy. Although the precise identity of these vesicles could not be discerned using this technique, their ~500 nm diameter is within the size range for early endosomes.^38^ Furthermore, the apparent lack of intraluminal vesicles and other intraluminal debris within these vesicles could indicate an early endocytic compartment. However, we cannot rule out the possibility that these vesicles represent a form of arrested lipophagy in the EtOH-damaged hepatocytes. In addition, although lipophagy has been shown previously to be preferential to small LDs ≤500 nm in diameter,^39^ Figure 6A suggests that even large LDs (2 μm) can be partially internalized by smaller endocytic vesicles. This process was not restricted to large LDs, however, as even small, 500 nm LDs appear to be engulfed by these vesicles (Figure 6B). Overall, these results suggest that while EtOH perturbs a downstream LD-lysosomal fusion pathway, the upstream process of LD-early endosome attachment may persist undeterred.

**FIGURE 7:**
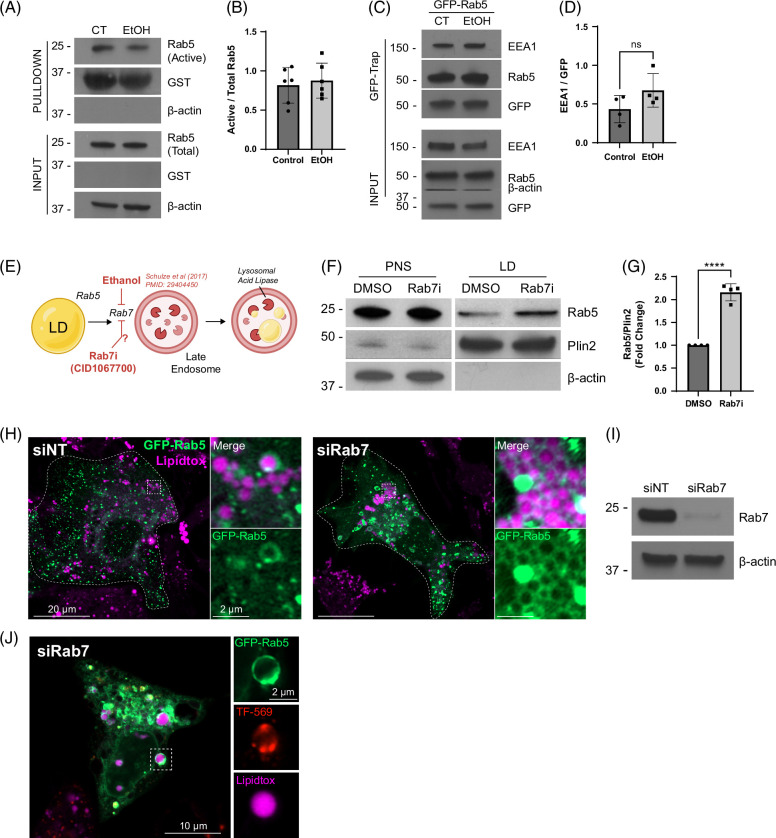
Inhibition of Rab7 increases Rab5-LD interactions. (A) Western blot showing GST pulldown of active Rab5 in primary hepatocytes from pair-fed control (CT) and EtOH-fed rats. (B) Densitometry quantification of active Rab5 from n=6 experiments. No measurable change in activity is observed. (C) Western blot of GFP-trap pull down from primary rat hepatocytes (CT vs. EtOH) expressing GFP-Rab5 to test for changes in the association with the Rab5 effector protein EEA1 by EtOH (D) Quantification of EEA1/GFP-Rab5 from n=4 experiments. (E) Cartoon depicting our hypothesis of a Rab5-to-Rab7 lipophagy pathway whereby EtOH inhibits Rab7 as described.^11^ (F) Western blot analysis of VA13 cells treated with DMSO carrier or the Rab7 inhibitor CID1067700 (24 h, 80 μM) showing an increase in Rab5-LD association. (G) Quantification of Rab5 abundance on isolated LDs from n=4 experiments supporting an increase in LD-associated Rab5 under Rab7 inhibition. (H) Confocal micrographs of control (siNT) versus Rab7 knockdown (siRab7) VA13 cells showing an increased GFP-Rab5 accumulation on LDs in Rab7 knockdown cells. (I) Representative western blot showing Rab7 knockdown efficiency. (J) Confocal micrograph of a Rab7-depleted VA13 cell showing LD surrounded by a Rab5 and transferrin-positive early endosome. Asterisks denote statistical significance by *t* test (*p < 0.05; **p < 0.01). Graphs depict mean and SD. Abbreviation: LD, lipid droplet.

As the increased LD-association of Rab5 in EtOH-damaged hepatocytes mimics that of cells expressing the S34N GTPase-defective Rab5 mutant (Figure 3), we reasoned that EtOH may inactivate the GTPase activity of this Rab. To test this premise, we utilized a previously described assay in which active Rab5 can be isolated from cell lysates using a GST fusion protein harboring the Rab5-binding domain of Rabaptin 5.^40^ This effector binds preferentially to active Rab5 and can be used to assess the activity of this GTPase in response to EtOH insult or other manipulations. To this end, GST-Rabaptin 5 pull downs were performed on primary hepatocyte cell lysates isolated from CT versus chronic EtOH-fed rats. Surprisingly, Rab5 activity was not significantly altered in chronic EtOH-fed hepatocytes (Figure 7A, B). To confirm this finding, Rab5 binding to its effector EEA1 was examined by expressing GFP-Rab5 in primary hepatocytes isolated from CT versus EtOH-fed rats that were then lysed for subsequent GFP-Rab5 isolation through GFP-Trap beads (Chroma). As active Rab5 is known to bind to its effector EEA1, GFP-Rab5 pull downs were then probed by western blot for associated EEA1 levels. No appreciable difference in EEA1-Rab5 association was observed from control versus EtOH-damaged hepatocytes. (Figure 7C, D) again suggesting that chronic EtOH exposure does not inhibit Rab5 GTPase activity.

**FIGURE 8:**
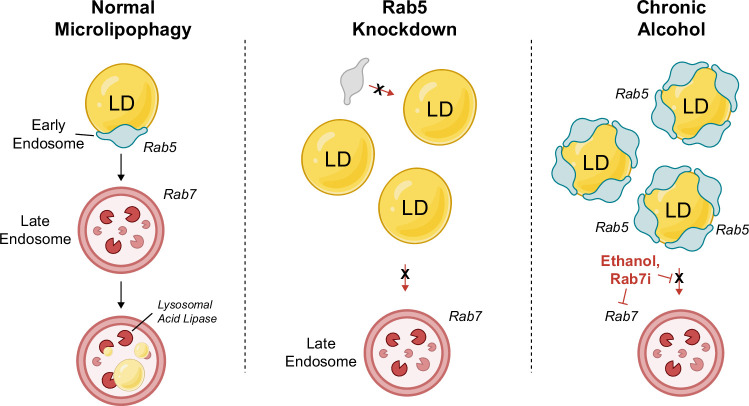
Rab5 cooperates with Rab7 during microlipophagy: a working model. Cartoon depicting microlipophagy as an endosomal process utilizing a Rab5-to-Rab7 transition that creates a favorable platform on the LD surface to facilitate LD-lysosome fusion/engulfment. This transition is inhibited by EtOH and responsible for decreased lipophagy and subsequent hepatic steatosis. Abbreviation: LD, lipid droplet.

From the unexpected results described above it became important to test if another endosome-based Rab protein might alter the distribution on LDs in response to EtOH. Our previous study had demonstrated that chronic EtOH exposure inhibits the GTPase activity of the MVB-associated Rab7 that is known to function downstream of Rab5.^11^ We reasoned that Rab7 downstream activity levels could impact upstream Rab5 localization to LDs as depicted in the cartoon in Figure 7E. To this end, we predicted that experimentally attenuating Rab7 with the Rab7 GTPase inhibitor CID1067700 (Rab7_i_), shown to bind competitively to the nucleotide binding pocket of Rab7,^41^ might alter Rab5 distribution is a way similar to an EtOH insult. Accordingly, VA13 cells were treated with DMSO or Rab7_i_ (80 μM, 24 h), followed by LD isolation using density gradient centrifugation. Western blot analysis revealed a significant 2-fold increase in Rab5 localization to LDs over DMSO controls (Figure 7F, G), suggesting that Rab7 GTPase activity influences Rab5 accumulation on LDs. As a second approach, siRNA knockdown of Rab7 in VA13 hepatocytes was performed in cells transfected with GFP-Rab5. Confocal micrographs of control (siNT) cells (Figure 7H) display normal, punctate distribution of GFP-Rab5 with some instances of LD localization. In contrast, Rab7-depleted cells (siRab7) displayed a GFP-Rab5 distribution that was transformed dramatically into a tubular morphology similar to that of EtOH-treated hepatocytes, with elevated localization to LDs. Furthermore, Rab7 knockdown caused the accumulation of Rab5 vesicles that were transferrin positive (Figure 7J, Supplemental Figure S5, http://links.lww.com/HC9/A892), suggesting that Rab7 mediates endolysosome maturation, but not LD-endosome engulfment. Taken together, these results are consistent with a model whereby EtOH inhibition of Rab7 disrupts an upstream, Rab5-dependent endosomal trafficking mechanism for LD-lysosome transport (Figure 8).

## DISCUSSION

The goal of this study was to provide new cellular and molecular insights into how the liver accumulates lipids in response to an EtOH insult. A seminal study from the Czaja group had implicated the canonical autophagic machinery in the catabolism of hepatocellular LDs.^5^ A second study from our group has indicated that a microlipophagic process is the predominant, but not exclusive, mechanism by which the hepatocyte catabolizes its LDs.^4^ In this process, the lysosome is targeted directly to the LD to engulf part or all of the LD. How this process is regulated and perturbed by EtOH exposure are central questions that need to be addressed. Specifically, how are lysosomes targeted to the LD surface and how is this interaction mediated? Clues to these events were provided by our observations in primary rat hepatocytes or VA13 hepatoma cells expressing alcohol dehydrogenase that revealed only modest lysosome-LD interaction in response to chronic or acute EtOH exposure thereby reducing access of the LD to the lysosomal lumen (Figure 1).

As lysosome transport and trafficking has been shown to be mediated in part by of Rab proteins, we examined the role of these small GTPases as EtOH targets in the LD-Lysosome docking process. Despite several LD proteomic studies demonstrating that Rab5 localizes to LDs,^18–23^ our understanding of its role in LD trafficking in cells, particularly in the context of liver diseases such as alcohol-associated steatosis, is limited. Here, we demonstrate for the first time that Rab5 mediates LD catabolism in hepatocytes, a process that is disrupted by chronic alcohol consumption. Moreover, Rab5 appears to be critical for LD-lysosome transport, perhaps independent of autophagosome-based lipophagy.

Rab5 has been shown to act as an early endosomal (EE) trafficking by directing fusion and maturation of EEs into MVBs. Recruitment of Rab5 to EEs is coincident with Rab5 activation into its GTP-bound state by guanosine exchange factors such as RABEX5. Activated Rab5 has several effectors that play diverse roles in EE trafficking. These include the effector linker protein EEA1 that acts to facilitate endosome-endos fusion, as well as PI3K which phosphorylates PI3P on EEs important to drive vesicle maturation. As EEs mature into more acidic MVBs and late endosomes, they typically lose their Rab5 signature in favor for Rab7, which is also an important driver of lipophagy in hepatocytes.^11,15^ In this manner, a Rab5-to-Rab7 transition marks the progression of endosomal cargo degradation. Thus we posit that Rab5, and its connection with Rab7, are central toward understanding how LDs enter the lysosome lumen.

Central to the findings of this study are our observations that Rab5 GTPase activity seems to play a role in regulating its recruitment to LDs. Both WT Rab5 and a constitutively active mutant form contact sites with the LD periphery, whereas the Rab5 inactive mutant (S34N) displays a more widespread subcellular distribution, perhaps around the endoplasmic reticulum. Most notable is that this S34N mutant becomes prominently associated with the LD surface (Figure 3). The biological relevance of this finding is currently unclear, as a previous study showed that treatment of cell lysates with GTPγS increased Rab5 association with LDs that were isolated by density gradient centrifugation. In addition, we find that expression of the WT and QL Rab5 proteins promote an association between LDs and transferrin-positive endosomes, while that of SN Rab5 does not (Figure 4, Supplemental Figure S3, http://links.lww.com/HC9/A892). This indicates that Rab5 recruits early or recycling endosomes to LDs depending on its GTP/GDP binding activity.

Our findings that Rab5 appears to recruit transferrin-positive endosomal components to the LD surface support a new perspective toward understanding the hepatocellular microlipophagic process with regard to lysosomal-LD interactions. Mainly, Rab5 directs an early endosome to the LD surface to act as a membranous platform intermediate between the 2 organelles to facilitate physical interactions and perhaps fusion. This intermediate would make sense in the context of canonical endocytosis processes in which lysosome fusion occurs, not with surface endocytic invaginations, but instead with subsequent endosomal intermediates. A Rab5-mediated association of an early endosome with a LD then makes its phospholipid monolayer into a pseudo-endosome and thus more “amenable” to lysosome docking.

How EtOH would impair this process is not fully clear to us as we do not observe direct reductions in Rab5 GTPase activity in primary hepatocytes using 2 distinct effector binding assays. This does not mean that reactive EtOH adducts could not alter one of the many guanine exchange factors, guanine dissociation factors, or GTPase activating proteins all known to regulate Rab function. Indeed, the finding that Rab5-LD interactions are altered by chronic EtOH is further supported by a recent LD proteomic study showing higher Rab5 levels on LDs from EtOH-fed rats.^23^ These findings are also consistent with the observations that EtOH consumption attenuates receptor-mediated endocytic trafficking^42^ as well as lipophagy by inhibiting Rab7 GTPase activity.^11^

Our findings that Rab5-LD association is increased by Rab7 inhibition, or siRNA knockdown (Figure 7), or in response to chronic EtOH consumption (Figure 5), suggest that the normal equilibrium between these 2 intimate Rab proteins is disrupted. The consequences of this could manifest in what we observe to be reduced lysosome-LD proximity and attenuated access of LDs to the lysosome lumen. Both of these disruptions contributing to what we have observed to contribute to EtOH-based hepatocyte steatosis. Further defining the detrimental effects of EtOH on Rab function and lipophagic membrane traffic as it contributes to increased hepatocellular LD content will prove important.

## Supplementary Material

SUPPLEMENTARY MATERIAL
