# Supplementary material for: Ethanol disrupts hepatocellular lipophagy by altering Rab5-centric LD-lysosome trafficking
Source: Hepatol Commun. 2024 May 22;8(6):e0446. doi: 10.1097/HC9.0000000000000446 (PMC11124685; doi:10.1097/HC9.0000000000000446)

Supporting Figure 1: LDs associate preferentially with endo-lysosomes versus autophagosomes. (A) Confocal images showing LD accumulation into lysosomes following LAListat in control cells (siNT), a process that is unaltered in cells with reduced Atg5 and FIP200. Cells were first loaded 2h with 150uM OA + 7.5μM Bodipy C12, then washed and chased an additional 24h in regular medium containing DMSO or 50μM LAListat prior to fixation. (B) Representative western blot showing Atg5 and FIP200 knockdown. (C) A fluorescence image of an OA-loaded VA13 cell expressing GFP-LC3 and co-labeled for LDs (MDH) and lysosomes (Lysotracker Deep Red), and treated with LAListat (24h, 50μM) shows LD accumulation within lysosomes with no LC3 present as depicted by white arrows. Graph depicts quantification of LD interactions observed with several degradative compartments that include lysosome (lysotracker only), autophagosomes (LC3 only), and autolysosomes (lysotracker+LC3) from >20 cells across n=3 experiments. LD-lysosome interactions are most abundant. (D) A fluorescence image of a Hep3B cell expressing GFP-LC3 and immunolabeled for the MVB marker protein CD63 (magenta) depicts examples of LD-CD63 association with no LC3 present. Graph depicts quantification of LDs following DMSO vs LAListat shows engulfment of LDs within CD63-positive endolysosomes, but not LC3 autophagosomes or CD63+LC3 amphisomes.

Supporting Figure 2: Rab5 mediates LD catabolism. Fluorescent images of VA13 cells tested for a role for Rab5 in LD catabolism in which cells are first lipid loaded with 150μM OA for 5h, followed by wash and 24h lipid withdrawal in the presence of DGAT1/2 inhibitors to prevent nascent LD synthesis. VA13 cells with depleted Rab5 retain substantially more LDs over time as quantitated in the adjacent bar graph depicting a greater that 80% loss of LDs in control siNT cells compared to a modest 30% loss in Rab5 depleted cells. Asterisks denote statistical significance by one-way ANOVA and Tukey's post hoc test (*, $P < 0.05$; **, $P < 0.01$). Graphs depict mean and SD.

Supporting Figure 3: LD-endosome interaction upon expression of GFP-Rab5 mutants. Companion to Figure 4a. (A) VA13 cell expressing constitutively active Q79L GFP-Rab5 showing GFP and transferrin-positive engulfment of LDs similar to that of WT GFP-Rab5 (see Fig. 4a), while cells expressing the dominant negative Rab5(S34N) show Rab5-coated LDs but with little to no association of a transferrin-containing endosome (B) suggesting this GTPase form may interact with the LD surface independent of an endosomal compartment.

Supporting Figure 4: LD-endosome interaction upon expression of GFP-Rab5 mutants. Companion to Figure 4b. Transmission electron micrographs showing additional examples of LD-endosome contacts in AML12 hepatocytes loaded 1h with Transferrin-Gold and starved in HBSS for 1h. White arrows depict transferrin-positive spots.

Supporting Figure 5: LDs accumulate within early endosomes in cells with reduced levels of Rab7. Confocal micrographs depict VA13 cells treated with Rab7 siRNA and transfected with GFP-Rab5. Cells were incubated for 30 mins with 25 µg/ml of transferrin-568 prior to fixation. The engulfment of LDs by Rab5-positive compartments are also positive for transferrin (red), suggesting these are early endosomes.

Supp. Figure 1

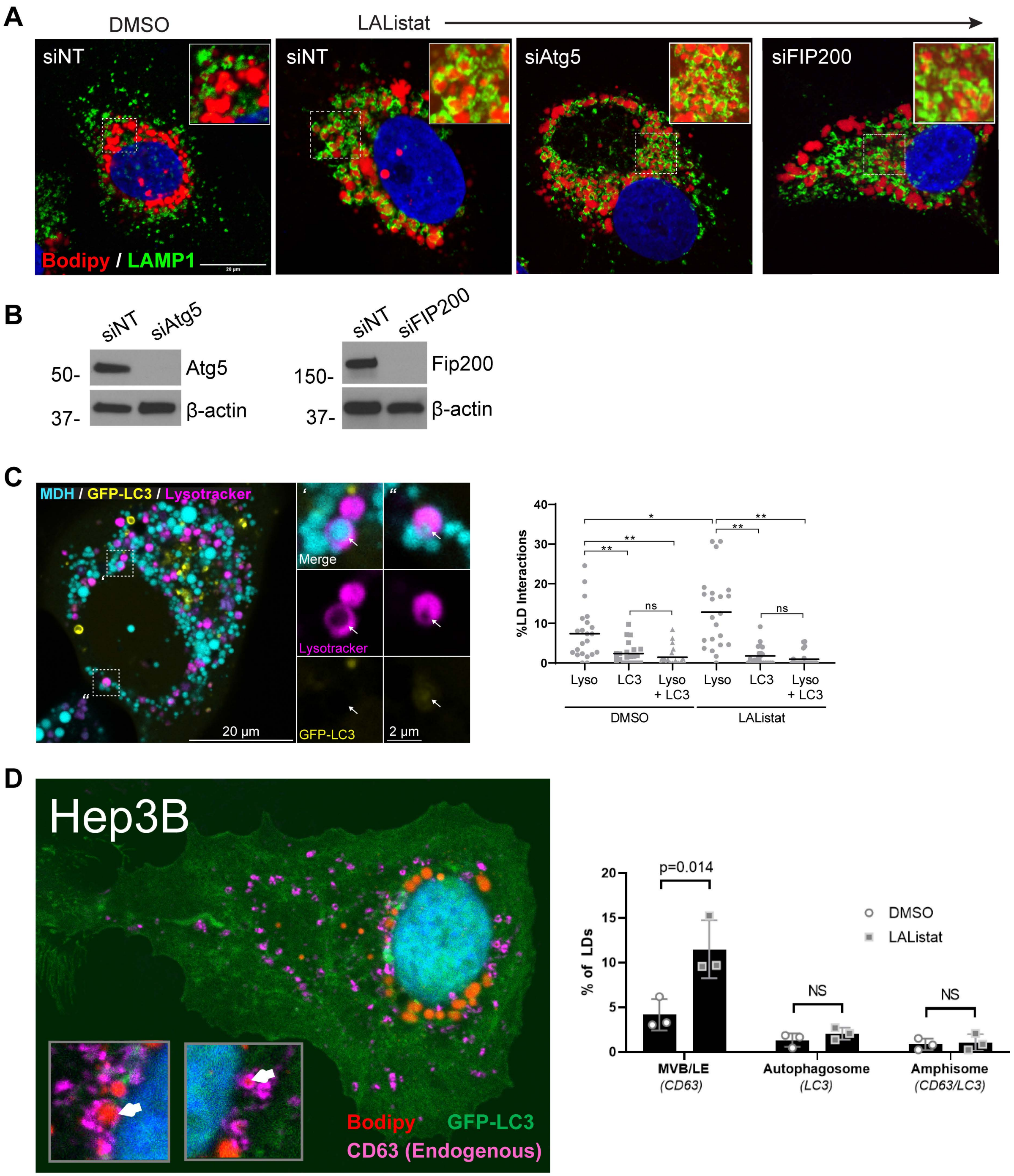

Supp. Figure 2

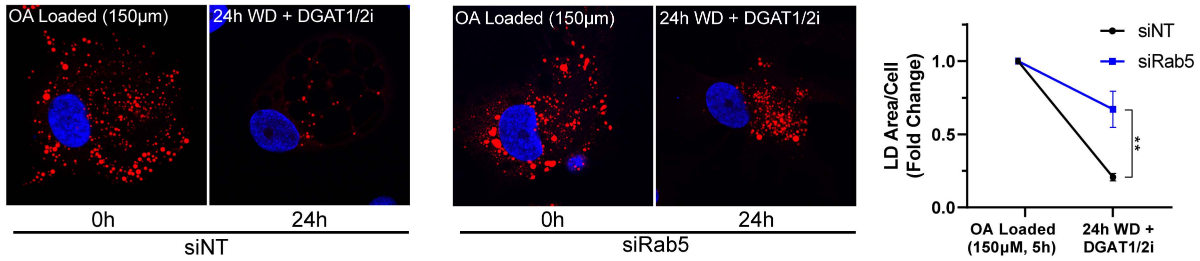

Supp Figure 3

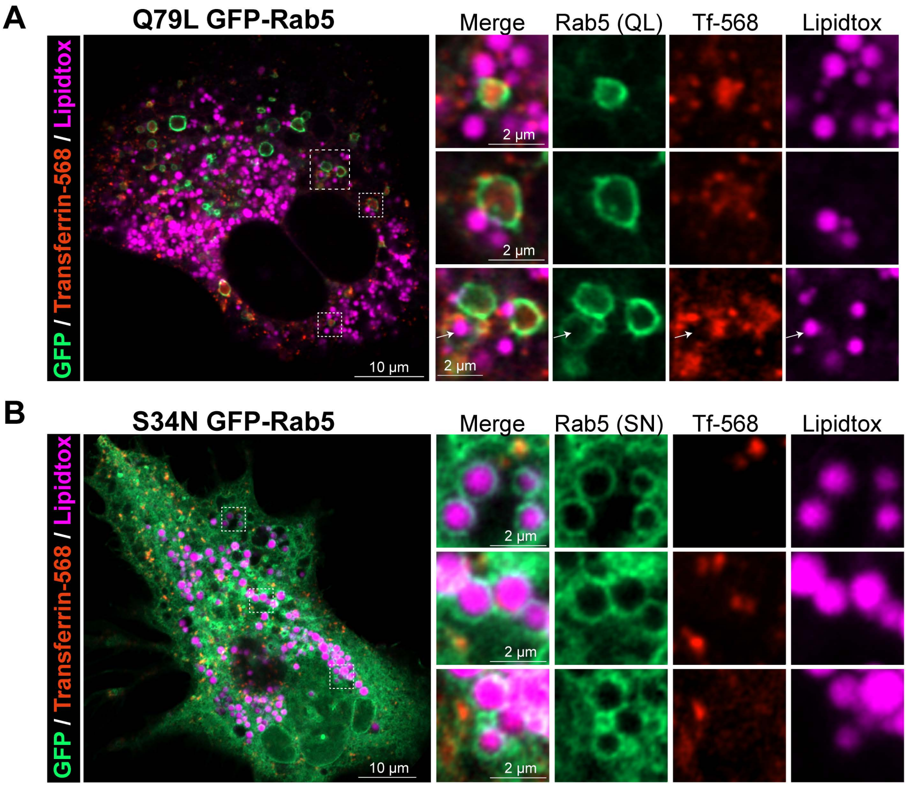

Supp. Figure 4

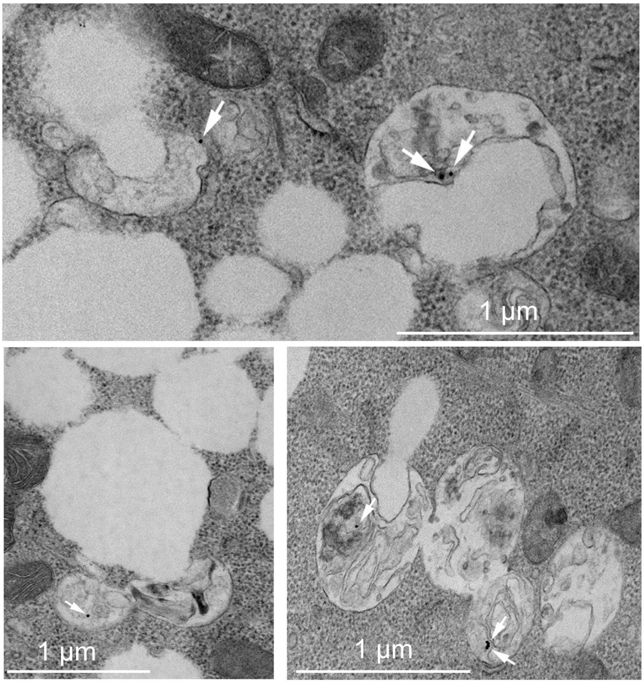

Supp. Figure 5

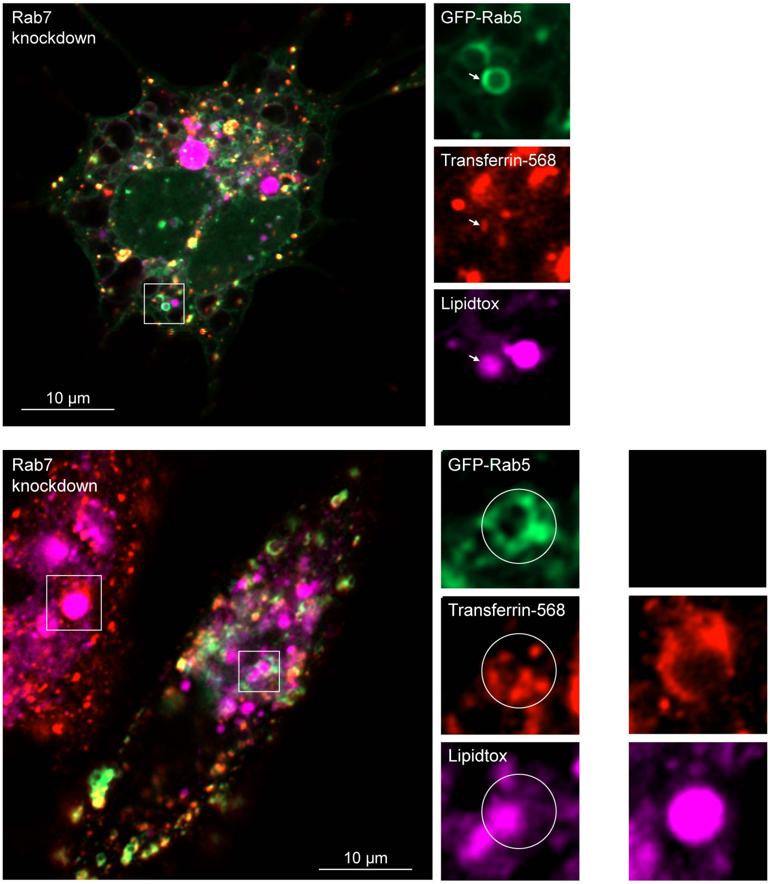

Supplement: SUPPLEMENTARY MATERIAL [file hc9-8-e0446-s001.pdf]
